# Mini-review: Lipid flippases as putative targets for biotechnological crop improvement

**DOI:** 10.3389/fpls.2023.1107142

**Published:** 2023-02-21

**Authors:** Rosa Laura López-Marqués

**Affiliations:** Department of Plant and Environmental Sciences, University of Copenhagen, Frederiksberg, Denmark

**Keywords:** flippases, P4 ATPases, resilience, stress adaptation, crop biotechnology

## Abstract

An increasing world population and drastic changes in weather conditions are challenging agricultural production. To face these challenges and ensure sustainable food production in the future, crop plants need to be improved to withstand several different biotic and abiotic stresses. Commonly, breeders select varieties that can tolerate a specific type of stress and then cross these varieties to stack beneficial traits. This strategy is time-consuming and strictly dependent on the stacked traits been genetically unlinked. Here, we revise the role of plant lipid flippases of the P4 ATPase family in stress-related responses with a special focus on the pleiotropic nature of their functions and discuss their suitability as biotechnological targets for crop improvement.

## Introduction

Agricultural production is facing enormous challenges. Yields must heavily increase to ensure enough food supply for an increasing world population (FAO, 2017). Simultaneously, this yield increase should be accompanied by a reduction in resource consumption and pollutant use, making food production more sustainable (Deleebeeck et al., 2021; Sarkar et al., 2021). However, the use of fertilizers and pesticides is key to maintaining yields in current agricultural production. Moreover, challenges such as extreme temperatures, heat and cold waves, drought, soil salinity, and the colonization of new habitats by plant pathogens complicate the transition to a high-yielding sustainable agriculture (Calicioglu et al., 2019; Jastrzębska et al., 2022).

In the past 15 years, enormous advances have been made in understanding the role of a family of lipid transporters (lipid flippases) in plant development and stress adaptation. Recent evidence suggests that these proteins could be interesting targets for breeding elite crops that are better at adapting to diverse environmental threats. In this review, we will recapitulate the existing information on plant flippase function and discuss it in the context of biotechnological crop improvement.

## Lipid flippases as active transporters

Most cellular membranes present an asymmetric lipid distribution between the two leaflets (transbilayer lipid asymmetry) that is essential for cell survival. Generation and maintenance of this asymmetry, at least for phospholipids, requires the action of lipid transporters that couple ATP hydrolysis to translocation of their preferred substrate(s) across the membrane (Visintainer and López-Marqués, 2021). These transporters are named flippases when the lipid is moved towards the cytosolic membrane leaflet, and floppases when the lipid is transported to the extracellular or lumenal side. Protein-dependent dissipation of the transbilayer lipid asymmetry is performed by ATP-independent scramblases that move lipids bi-directionally following their concentration gradient in the membrane (Visintainer and López-Marqués, 2021).

Inward-transporting lipid flippases mainly belong to the superfamily of P-type ATPases, which encompasses active transporters that form a phosphorylated intermediate during catalysis (Palmgren and Nissen, 2011). P-type ATPases can be divided into 5 subfamilies according to substrate specificity, of which the P4-ATPase family comprises lipid transporters (Palmgren and Axelsen, 1998). In plants, P4 ATPases are commonly known as AminophosphoLipid ATPase (ALA) proteins. This nomenclature can be deceiving, since several ALAs can transport phospholipids that do not contain amino groups in their head group, and at least one can recognize lipids without a phosphate group (see below).

Arabidopsis has 12 P4 ATPases (ALA1−ALA12), several of which have been characterized with respect to their transport specificity and physiological function (**Table 1**). ALA1 was the first Arabidopsis P4 ATPase to be described. Through heterologous expression in a yeast mutant devoid of an endogenous P4 ATPase (*Δdrs2*), ALA1 was shown to be able to translocate a fluorescent analog of phosphatidylserine (PS) (Gomes et al., 2000). Later, ALA1 failed to transport natural PS when expressed in another yeast strain lacking 3 endogenous P4 ATPases (*Δdrs2Δdnf1Δdnf2*), but an alternative substrate could not be identified (López-Marqués et al., 2012). Interestingly, the closest mammalian homologs of ALA1 are ATP10A and ATP10D (Palmgren et al., 2019), which have been shown to transport phosphatidylcholine (PC) and the glycosphingolipid glucosylceramide (GlcCer), respectively (Naito et al., 2015; Roland et al., 2019). Whether ALA1 shares a substrate preference with one of the mammalian ATP10 proteins remains to be tested. ALA2 seems to be highly specific and recognizes exclusively PS. However, only the 3 major phospholipids (phosphatidylserine (PS), phosphatidylcholine (PC) and phosphatidylethanolamine (PE)) were tested (López-Marqués et al., 2010). In contrast, ALA3, ALA5, and ALA10 appear to have a broad specificity. ALA3 transports PS and PE at almost equal rates, and can also recognize PC (Poulsen et al., 2008; López-Marqués et al., 2010). Further work showed ALA3-dependent phosphatidylglycerol (PG) transport but failed to detect significant PE and PC translocation (Jensen et al., 2017). ALA5 has a preference for PC, but still recognizes PE to a significant extent in fluorescent lipid-based uptake assays (Davis et al., 2020). In addition, yeast expression of ALA5 results in resistance to low concentrations of the toxic PS-binding peptide papuamide, indicating that the plant protein can also transport natural PS (Davis et al., 2020). In the same experiments, ALA5 was shown to transport ceramide-derived sphingomyelin. Since this lipid is only present in animals, it is most likely not a true ALA5 substrate, and its recognition is probably due to the presence of a phosphocholine head group equivalent to that of PC. While ALA10 has a preference for PC (and SM), it can also transport PS, PE, lysoPC, PG, and smaller amounts of non-phosphate containing glucosylceramide (Poulsen et al., 2015; Jensen et al., 2017). Using the same yeast-based fluorescent lipid uptake assays applied to other ALA proteins, attempts were made to identify a lipid substrate for ALA6, but these experiments were unsuccessful, probably due to the lack of an unknown cofactor (McDowell et al., 2015). However, only PS, PE, PC, and the minor signaling phospholipid phosphatidic acid (PA) were tested. With the current knowledge, these experiments should be revised to include at least PG and GlcCer. The transport capabilities of the other 6 Arabidopsis P4 ATPases (ALA4, ALA7, ALA8, ALA9, ALA11 and ALA12) are yet to be tested. Lipid uptake assays for ALA proteins from plants different from Arabidopsis have not been reported yet.

**Table 1:** Substrate specificity and physiological functions of plant P4 ATPases.

P4 ATPase	Relevant substrate(s)	Mutant phenotypes	Effect of overexpression	References
*Arabidopsis thaliana*				
ALA1	(PS)*	-Chilling tolerance -Defective antiviral defence -Defective mycotoxin detoxification	In maize: -Increased resistance to *F. graminearum* -Reduced mycotoxin accumulation	(Gomes et al., 2000; López-Marqués et al., 2012; Guo et al., 2017; Zhu et al., 2017; Wang et al., 2021)
ALA2	PS	-Defective antiviral defence	In yeast: -Heavy metal tolerance	(López-Marqués et al., 2010; Guo et al., 2017; Zhu et al., 2017)
ALA3	PS=PE>PG>PC	-Abnormal trichome branching -Nutrition- and temperature-dependent growth defects -Impaired reproductive development -Impaired auxin-mediated development -Defective fungal defence responses -Defective gravitropism -Defective pollen tube growth and guidance	n.a.	(Poulsen et al., 2008; Zhang and Oppenheimer, 2009; López-Marqués et al., 2010; McDowell et al., 2013; Zhang et al., 2020; Zhou et al., 2020; Yang et al., 2022)
ALA4	Not tested	-Heavy metal sensitivity -Dwarfism (*ala4 ala5* double mutant)	n.a.	(Sanz-Fernández et al., 2017; Davis et al., 2020)
ALA5	PC>PE>PS	-Dwarfism (*ala4 ala5* double mutant)	n.a.	(Davis et al., 2020)
ALA6	No transport detected	-heat sensitivity -Defective pollen tube growth (*ala6 ala7* double mutant) -Impaired fertility (*ala6 ala7* double mutant)	-Heat tolerance	(McDowell et al., 2015; Niu et al., 2017)
ALA7	Not tested	Defective pollen tube growth (*ala6 ala7* double mutant) -Impaired fertility (*ala6 ala7* double mutant)	n.a.	(McDowell et al., 2015)
ALA10	PC>PE=PS>PG= lysoPC>GlcCer	-Defective stomatal movements -Defective phosphate starvation responses -Altered chloroplast PC homeostasis	-Chilling tolerance	(Poulsen et al., 2015; Botella et al., 2016; Jensen et al., 2017)
*Oryza indica*				
YLD1	Not tested	-Premature senescence -Dwarfism	n.a.	(Deng et al., 2017)
*Gossypium hirsutum*				
GbPATP	Not tested	-Chilling sensitivity	In tobacco: -Chilling tolerance	(Liu et al., 2015)

*ALA1 was initially shown to transport fluorescent PS, but natural PS transport could not be observed in a later report.

PS, phosphatidylserine; PC, phosphatidylcholine; PE, phosphatidylethanolamine; PG, phosphatidylglycerol; GlcCer, Glucosylceramide; n.a., not available.

Lipid transport has been tested by yeast heterologous expression in all cases, except for ALA10, for which transport of fluorescent NBD-PC, -PE, -PS and -lysoPC were also demonstrated in planta. Only PS transport by ALA3 and lysoPC transport by ALA10 have been directly linked to the phenotypes observed in mutant plants (see main text).

## Molecular functions of lipid transport by P4 ATPases

As a consequence of their lipid transport activity, plant P4 ATPases have molecular functions in formation of vesicles, recruitment of lipid-binging proteins, and lipid signaling. For instance, ALA3 is involved in formation of vesicles in leaves, roots and growing pollen tubes (Poulsen et al., 2008; McDowell et al., 2013; Underwood et al., 2017; Zhang et al., 2020). It has been postulated that the capacity of P4 ATPases to pump lipids results in an imbalance in the lipid numbers between the two leaflets that forces the membrane to bend, initiating vesiculation (Tannert et al., 2003). In addition, ALA3 is capable of interacting both physically and genetically with small GTPases involved in the first steps of vesicle formation (Zhang et al., 2020; Zhou et al., 2020; Yang et al., 2022). Since generation of membrane curvature has never been directly demonstrated for any P4 ATPase, the question remains whether ALA3 contributes to vesicle formation by bending the membrane or simply by facilitating the recruitment of vesicle-related proteins, either through direct interaction or through local accumulation of PS. In fact, several of the aforementioned small GTPases are capable of directly binding anionic lipids, including PS, which is a substrate for ALA3 (Zhou et al., 2020). In the growing pollen tube of wild-type plants, ALA3 activity results in an asymmetric subcellular distribution of PS, which accumulates in the apical zone (Zhou et al., 2020). This asymmetric PS distribution seems essential for correct localization of the small GTPases, and the proteins are mislocalized in *ala3* mutant plants, where PS is also abnormally distributed (Zhou et al., 2020). However, the tested small GTPases bind phosphoinositides (PIPs) more effectively than PS *in vitro*, and it cannot be ruled out that a change in the PIP distribution is the underlying cause for the observed mislocalization in *ala3* plants. Trafficking of pollen-specific receptor kinases (PRKs) to the tip of the growing pollen tube was also linked to ALA3-dependent apical accumulation of PS (Yang et al., 2022). Several PRKs contain PS-binding polybasic motifs, and their elimination results in protein mislocalization, indicating that direct binding to PS is important for PRK trafficking (Yang et al., 2022). Other plant flippases, such as ALA1 and ALA7 are also involved in membrane trafficking (Wang et al., 2021), but their action mechanisms are unknown. The only confirmed example of a plant flippase involved in lipid signaling is ALA10, which was shown to be required for lysoPC signaling during light-dependent stomatal movement and adaptive responses to phosphate starvation (Poulsen et al., 2015). A role in GlcCer signaling was proposed for ALA4 and ALA5, but transport of this lipid could not be confirmed for ALA5, and ALA4 was not tested (Davis et al., 2020).

## Potential use of P4 ATPases as biotechnological tools for crop improvement

**Figure 1:**
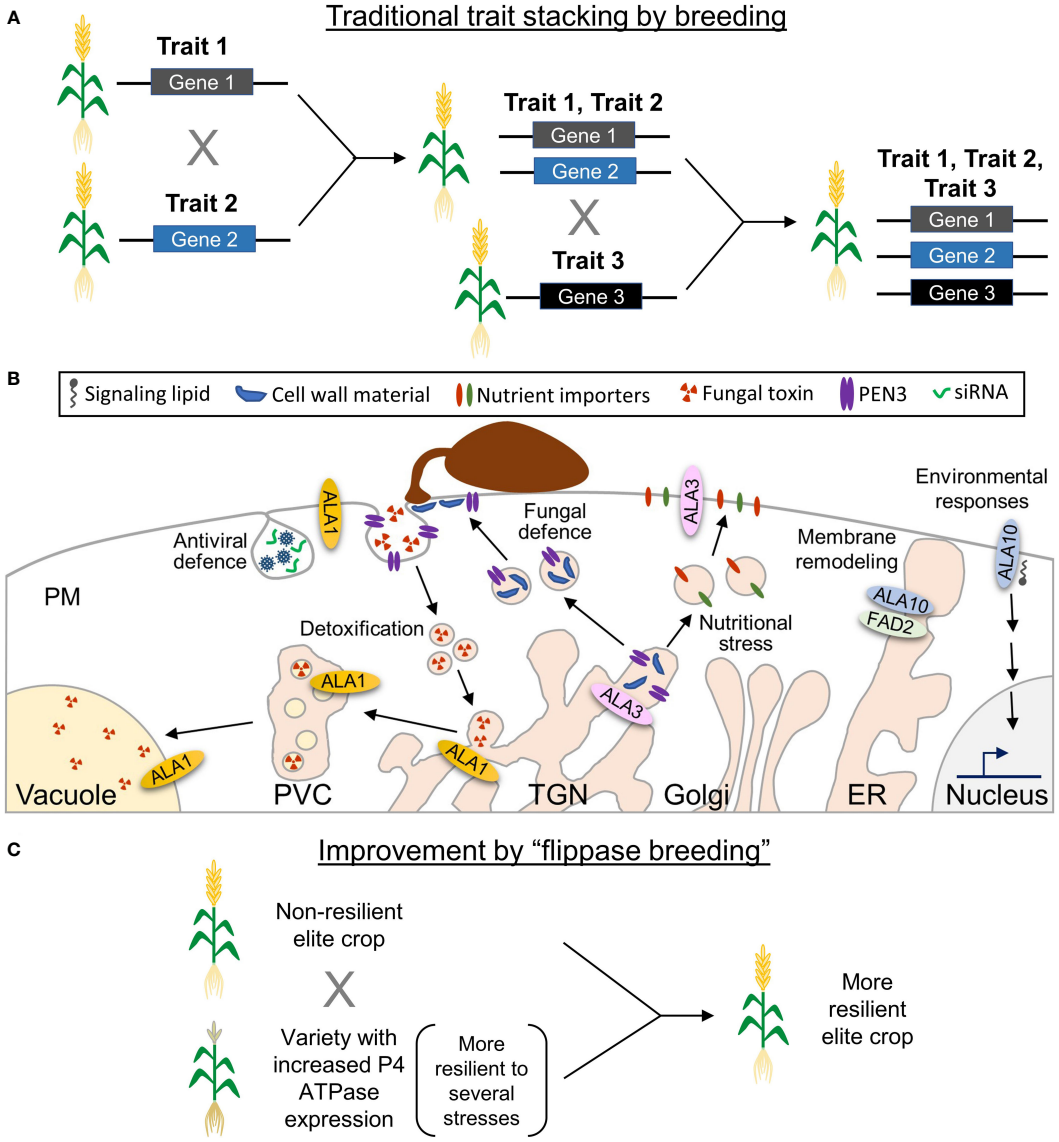
Potential of lipid flippases as biotechnological targets for crop improvement. **(A)** In traditional breeding strategies, generation of plants with several desirable traits is accomplished by successively crossing plants that contain each of the individual traits, which makes the process time-consuming. Moreover, due to the chromosomal position of some of the corresponding genes, the stacking of some traits might not be possible. **(B)** Some Arabidopsis P4 ATPases show pleiotropic functions. ALA1 is involved in small interference (si)RNA-based antiviral defense and detoxification of fungal metabolites. ALA3 was suggested to contribute to trafficking of cell wall material and the ABC transporter PENETRATION (PEN)3 during antifungal responses, as well as to trafficking of ion importers required for adaptation to nutritional challenges. ALA10 controls membrane fluidity during cold stress probably through its interaction with FATTY ACID DESATURASE (FAD)2 and contributes to lysoPC signaling events during adaptation to environmental cues, such as light-activated stomatal opening and phosphate starvation responses. **(C)** Based on pleitropic functions assigned to lipid flippases, it is possible to hypothesize that plants with a higher P4-ATPase expression will be more resilient to several types of stress. Crossing these plants with elite crop varieties would directly result in elite crops with enhanced resilience.

Climate changes in the past decades have resulted in extreme weather conditions worldwide, with higher temperatures and frequent heat waves interspersed with heavy rainfall periods leading to severe floodings (Pörtner et al., 2022). This weather has affected the ecosystems and resulted in shifts in the geographic range and seasonal behavior of several species, favoring the spreading of disease vectors (vira, bacteria, fungi), including plant pathogens, to previously uncolonized habitats (Pörtner et al., 2022). Future crops will therefore need to be resilient to the emerging multicomponent challenges, while still maintaining their productivity. In this context, crop biotechnology and advanced breeding strategies will be instrumental to ensure sustainable and sufficient agricultural production. The most pressing challenge is to identify the best genetic targets for crop improvement. Many crop improvement strategies aim at enhancing resilience to one specific type of stress (pathogens, temperature, drought, salinity). Then, plants are crossed to obtain new varieties that stack several desirable traits (**Figure 1A**). This process is slow and depends on the desired traits being genetically compatible with each other (Chen and Ow, 2017; Kumar et al., 2017). Thus, a target that allows plants to mount an effective response to more than one kind of stress simultaneously would be an enormous advantage. Unfortunately, almost nothing is known about the functions of P4 ATPases in crops. In Arabidopsis, they are crucial for plant adaptation to several biotic and abiotic challenges, and at least some of them show pleiotropic functions in stress tolerance (**Figure 1B**), which would make these proteins ideal candidates for effective crop improvement. In the following, we will focus on the information available for the Arabidopsis P4 ATPases, unless otherwise indicated.

The best example of a putative flippase target is probably ALA10. Efforts to generate drought tolerant crops have often focused on the reduction of stomatal numbers to reduce transpiration and increase water use efficiency, even at the cost of photosynthesis (Lawson and Blatt, 2014; McAusland et al., 2016). It was proposed that an increase in the speed of the stomatal movements, which are delayed with respect to photosynthesis, could be beneficial to obtain effective crops with higher drought resistance (Lawson and Blatt, 2014). Arabidopsis plants lacking ALA10 show a defect in stomatal opening and closure that likely results from lack of lysoPC signaling in guard cells (Poulsen et al., 2015). This defect results in reduced transpiration both in light and darkness (30 – 40%), without a decrease in net photosynthesis, which suggests that ALA10 removal might be a good strategy to generate plants with higher water use efficiency without a growth penalty. Interestingly, the *ALA10* gene is upregulated around 20% during phosphate starvation and *ala10* mutant plants show a defect in lysoPC signaling in roots under these conditions (Poulsen et al., 2015). Unfortunately, ALA10-overexpressing lines were not generated in these experiments, so the possibility that increased expression of ALA10 might result in drought and phosphate-deficiency tolerant plants remains to be tested. ALA10 overexpressing lines were tested for growth under chilling conditions and shown to be better at adapting to cold temperatures than wild-type plants, probably due to more effective cooperation of ALA10 with the fatty acid desaturase FAD2, which alters the FAD2/FAD3 balance affecting membrane fluidity (Botella et al., 2016). Finally, although the involvement of ALA10 in viral defenses was not tested, ALA10 expression levels increase about 2-fold when wild-type plants are treated with cucumber mosaic virus (Zhu et al., 2017), which suggests a yet undefined role for the ALA10 protein in viral defenses. Thus, a breeding strategy resulting in increased expression of the ALA10 homolog in crops might result in beneficial pleiotropic effects and plants that are simultaneously better at adapting to chilling temperatures, phosphate scarcity and water deprivation, while putatively being more resistant to viral attacks.

Another plant flippase with demonstrated pleiotropic functions in stress tolerance is ALA3. *ala3* plants show growth defects that are dependent on the nutritional conditions (McDowell et al., 2013). ALA3 seems to have a key role in vesicle formation in the secretory pathway and these defects are probably due to two overlapping effects: 1) the lack of mucilage secretion, which will affect the solubility of important nutrients in the soil, e.g. phosphate (Poulsen et al., 2008); 2) the defective trafficking of membrane transporters to the plasma membrane of root epidermal cells, which will affect nutrient uptake (McDowell et al., 2013). While trafficking of nutrient uptake systems to the plasma membrane has not been tested in Arabidopsis *ala3* plants, PIN-FORMED (PIN) auxin transporters are mislocalized in the roots of these mutant plants, similarly to specific plasma-membrane receptor kinases in the pollen tube (Zhang et al., 2020; Zhou et al., 2020; Yang et al., 2022) and the fungal defense-related ABC-transporter PENETRATION (PEN)3 in leaves (Underwood et al., 2017). The growth defects of *ala3* plants are worsened by both low and high temperatures, and significant differences in rosette diameter could be detected when the temperature was lowered from 24 to 20°C (McDowell et al., 2013). A similar effect was observed on root growth when the temperature was lowered from 26 to 23°C or increased to 30°C, which suggests that ALA3 function is important for temperature adaptation. As for ALA10, overexpression of ALA3 was not tested, but it might result in plants with enhanced nutrient uptake, better temperature adaptation capabilities, and faster responses to pathogens.

A final example of a P4 ATPase with pleiotropic stress-related functions is ALA1, which was first linked to chilling tolerance. Whether this is due to interaction with desaturases, in a manner analogous to that of ALA10, or by another mechanism is not known (Gomes et al., 2000). Interestingly, an ALA1 homolog in cotton (GbPATP) is also involved in chilling tolerance and overexpression of the cotton gene confers cold resistance to *Nicotiana tabacum* plants (Liu et al., 2015), suggesting that flippase functions might be conserved across plant species. ALA1 is also necessary for viral gene silencing during infection in Arabidopsis (Guo et al., 2017; Zhu et al., 2017). While direct prove of the molecular mechanism was not provided, it was suggested that ALA1 is necessary for generating membrane invaginations that are required for encapsulation of the viral replication machinery during antiviral responses (Guo et al., 2017). Finally, loss of ALA1 prevents proper detoxification of the fungal toxin deoxynivalenol (DON), which is produced by *Fusarium graminearum*, the main agent of Fusarium Head Blight in cereals (Wang et al., 2021). In this case, lack of proper trafficking seems to be the underlying cause of the detoxification defect. Notably, overexpression of Arabidopsis ALA1 in maize resulted in enhanced resistance to *F. graminearum* and decreased accumulation of the mycotoxin (Wang et al., 2021), which makes ALA1 homologues very promising targets to generate fungal disease resistant crops. Considering the aforementioned results on GbPATP, such crops would also be expected to show chilling tolerance.

While possible pleiotropic functions have not been described for other plant flippases, some still show stress-related functions that might be interesting from the point of view of biotechnological crop improvement. For instance, *ala2* plants show defects in antiviral defenses (Guo et al., 2017; Zhu et al., 2017). ALA6 is involved in adaptation to heat stress and *ALA6* overexpression results in increased heat tolerance, probably due to enhanced plasma membrane properties and reduced leakage (Niu et al., 2017). Finally, *ALA4* expression is upregulated in the presence of heavy metal ions and plants lacking ALA4 are sensitive to heavy metals (Sanz-Fernández et al., 2017).

## Conclusion

The study of P4 ATPases in crops is in its infancy and only two reports have so far dealt with this subject: a report on the involvement of GbPATP in chilling tolerance (Liu et al., 2015) and a study showing the role of YELLOW LEAF AND DWARF (YLD)1 in senescence in rice (Deng et al., 2017). Nonetheless, accumulating evidence in Arabidopsis suggests that P4 ATPases are relevant targets for crop improvement based on their involvement in plant adaptive responses to different types of biotic and abiotic stresses. These include viral and fungal diseases, which have been estimated to cause crop losses of 20–40% of the global production (Savary et al., 2012), as well as temperature challenges, which are expected to worsen in the future (Pörtner et al., 2022). Unfortunately, most of our knowledge comes from P4-ATPase gene knockouts in Arabidopsis and analysis of the corresponding loss-of-function mutants. These mutants show detrimental effects, but whether enhanced expression of the genes will result in plants better suited to withstand environmental challenges is mostly unknown. However, there are a few very interesting examples, in which overexpression of a flippase, such as ALA6 or ALA10 results in Arabidopsis plants less sensitive to extreme temperatures (heat and chilling, respectively). Most notably, overexpression of Arabidopsis ALA1 in maize confers resistance to fungal infections, while overexpression of a cotton homologue of ALA1, GbPATP, in tobacco results in plants with chilling tolerance. These results suggest that at least some important stress-related functions of the Arabidopsis proteins are conserved in crops of agricultural relevance. We previously hypothesized that the positive effect of P4 ATPases in stress adaptation might not be related to their involvement in specific cellular processes, but rather be the consequence of the general function of these transporters in maintaining membrane properties, facilitating membrane trafficking, and participating in effective lipid signaling (Nintemann et al., 2019). If this were true, crop varieties with enhanced expression of P4 ATPases should be more effective at responding and adapting to several types of biotic and abiotic stresses simultaneously. This opens for the possibility of generating new resilient varieties for agricultural production by simple crossing of elite crops with landraces with high P4-ATPase expression (**Figure 1C**). Considering the putative impact in ensuring future food production, upcoming studies on the physiological functions of plant P4 ATPases should focus on understanding the role of these transporters in crop stress tolerance and testing the validity of this hypothesis.

## Author contributions

RLM researched the literature, interpreted, and conceptualized the data, and wrote the manuscript. RLM approves the final version of the manuscript, ensures the accuracy and integrity of the work, and agrees to be accountable for all aspects of the work. The author confirms being the sole contributor of this work and has approved it for publication.
